# Synthesis, biological activities and docking studies of pleuromutilin derivatives with piperazinyl urea linkage

**DOI:** 10.1080/14756366.2021.1900163

**Published:** 2021-03-18

**Authors:** Yuanyuan Zhang, Chuan Xie, Yang Liu, Feng Shang, Rushiya Shao, Jing Yu, Chunxia Wu, Xinghui Yao, Dongfang Liu, Zhouyu Wang

**Affiliations:** a Department of Chemistry, School of Science, Xihua University, Chengdu, China; b Yibin Key Laboratory of Research and Application of Small Organic Chiral Molecules, Yibin Research Institute of Xihua University, Yibin, China; c Kampo Medicine Pharmacology Research Laboratory, Graduate School of Pharmaceutical Sciences, Yokohama University of Pharmacy, Yokohama-shi, Japan

**Keywords:** Pleuromutilin, piperazinyl urea, synthesis, biological activities, MRSA

## Abstract

Antibiotics resistance is becoming increasingly common, involving almost all antibiotics on the market. Diseases caused by drug resistant bacteria, such as MRSA, have high mortality and negatively affect public health. The development of new drugs would be an effective means of solving this problem. Modifications based on bioactive natural products could greatly shorten drug development time and improve success rate. Pleuromutilin, a natural product from the basidiomycete bacterial species, is a promising antibiotic candidate. In this study, a series of novel pleuromutilin derivatives possessing piperazinyl urea linkage were efficiently synthesised, and their antibacterial activities and bactericidal properties were evaluated via MIC, MBC and Time-kill kinetics assays. The results showed that all compounds exhibited potent activities against tested strains, especially MRSA strains with MIC values as low as 0.125 μg/mL; 8 times lower than that of marketed antibiotic Tiamulin. Docking studies indicate substituted piperazinyl urea derivatives could provide hydrogen bonds and initiate π-π stacking between molecules and surrounding residues.

## Introduction

Current available antibiotics are losing their effectiveness due to increasing bacterial resistance. Diseases caused by multi-drug resistant bacteria are particularly difficult to prevent and treat, and across the world, approximately 700,000 people die annually as a result of them^1^. Among drug-resistant strains, methicillin-resistant *Staphylococcus aureus* (MRSA) is one of the most problematic, with pathogens that spread rapidly and cause severe diseases such as septicaemia, pneumonia, osteomyelitis and endocarditis^2^^,^^3^. Average treatment time and MRSA patient mortality are, respectively, five and three times higher than those of patients infected by common pathogens. Antibiotics are generally used to control MRSA infections, however the majority of common antibiotics used in clinical treatment were ineffective against MRSA. Therefore, it is crucial to develop and discover novel antibacterial agents^4^.

**Scheme 1.**
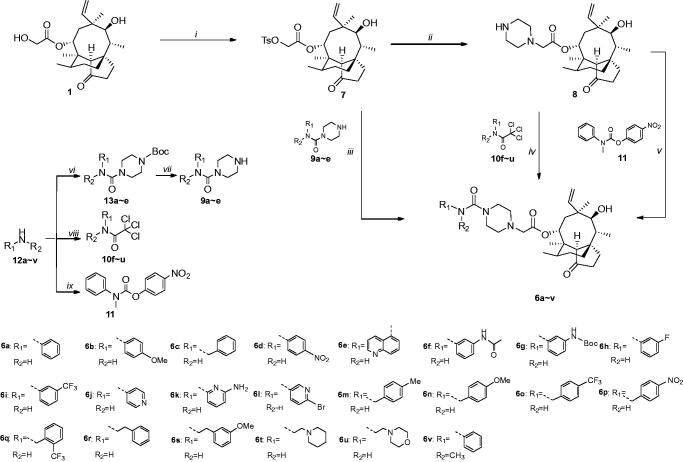
Synthesis route of target compounds **6a ∼ v**. Reagents and conditions: (*i*) TsCl, triethylamine, DCM, 25 °C; (*ii*) Piperazine, K_2_CO_3_, NaI, THF, reflux; (*iii*) **9a ∼ e**, NaI, K_2_CO_3_, MeCN, 70 °C; (*iv*) **10f ∼ u**, K_2_CO_3_, DMF, 80 °C; (*v*) **11**, DMAP, MeCN, 70 °C; (*vi*) CDI, triethylamine, N-Boc piperazine, MeCN and DMF, 25 °C for **13a** and **13b**, or BTC, triethylamine, N-Boc piperazine, THF and DCM, 25 °C for **13c ∼ e**, (*vii*) TFA, DCM, 25 °C; (*viii*) Trichloroacetyl chloride, triethylamine, DCM, 25 °C; (*ix*) 4-Nitrophenyl carbonochloridate, NMM, DCM, 25 °C.

**Figure 1.**
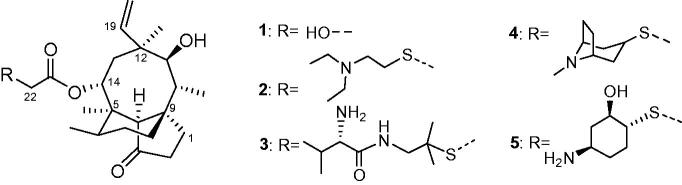
Structures of pleuromutilin derivatives **2** ∼ **5** developed from natural Pleuromutilin **1**.

**Figure 2.**
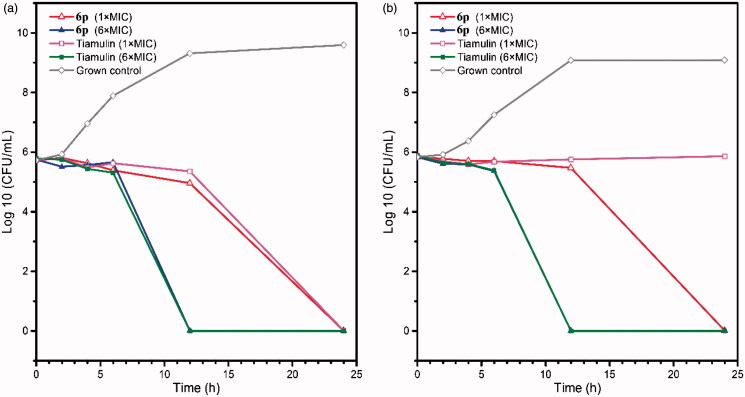
Time-kill kinetics of compound **6p** against MRSA ATCC 33591(**a**) and *E. coli* ATCC 25922 (**b**).

**Figure 3.**
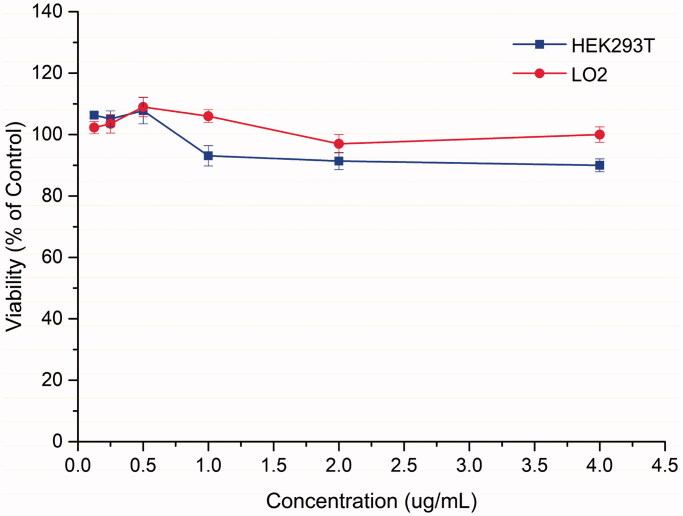
Effects of compound **6p** on cell viability by CCK-8 assay.

**Figure 4.**
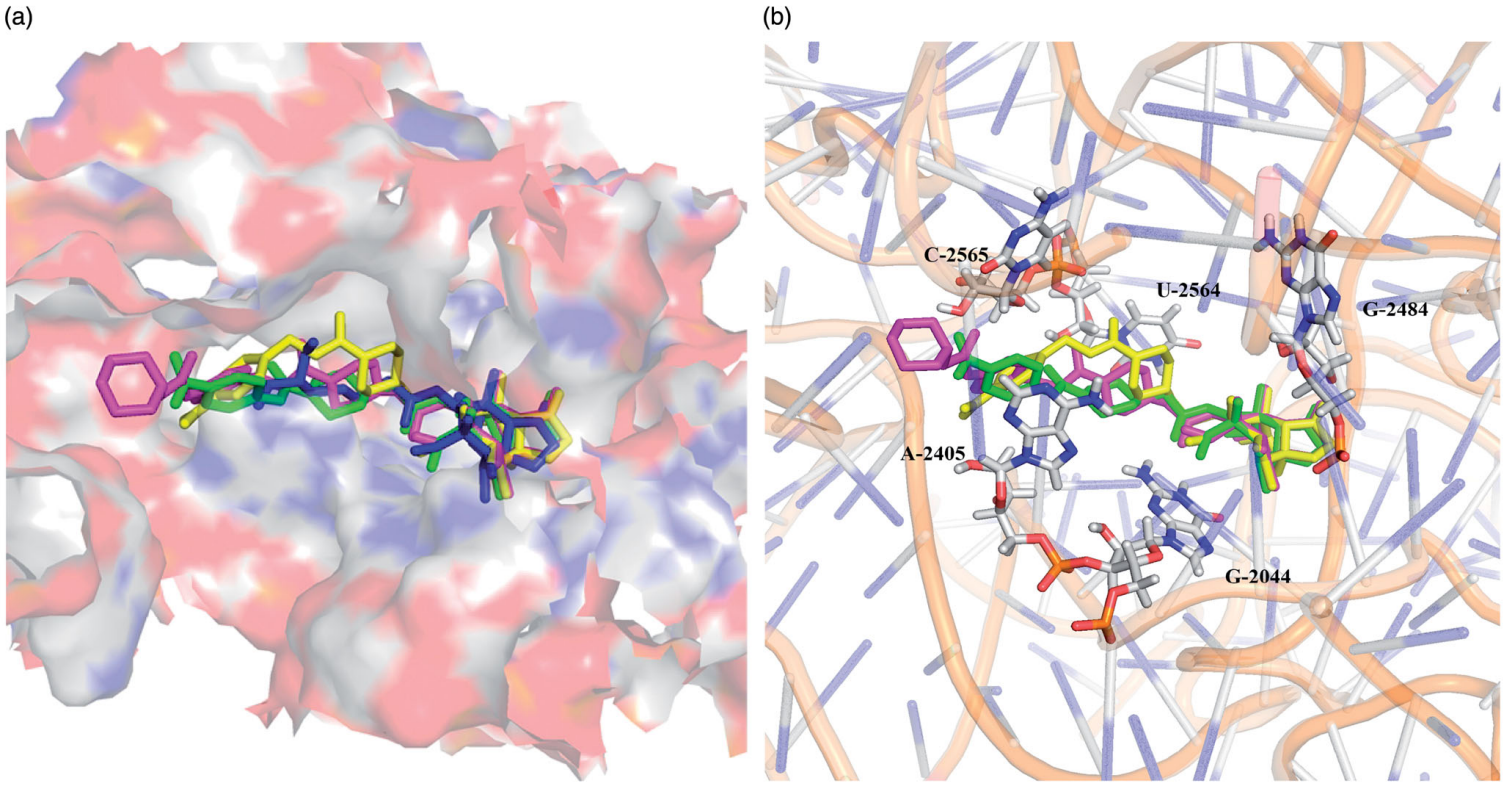
Superimposed poses of selected ligands in the PTC of the 50 s ribosomal subunit. (**a**) The interaction groove; (**b**) Residues involved in the interactions between ligands and PTC.

**Figure 5.**
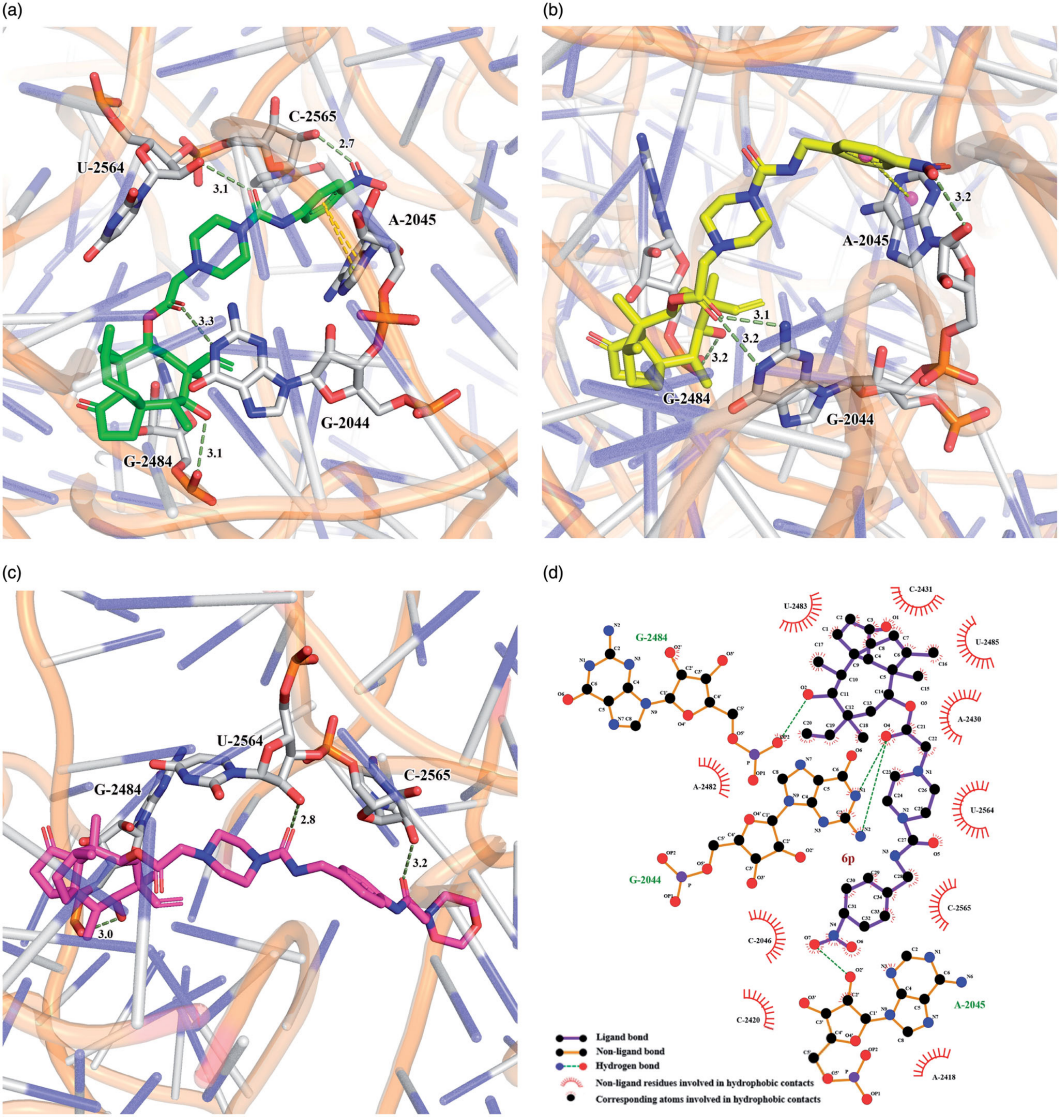
Interactions between ligands and residues of the 50 s ribosomal subunit. (**a**) **6d** and site; (**b**) **6p** and site; (**c**) **6z** and site; (**d**) Hydrogen bonds and hydrophobic interactions.

Natural products such as Penicillin, Erythromycin, Cephalosporin C and Kanamycin are often used as main compounds in drug discoveries due to their potent bioactivity. Corresponding semi-synthesised derivatives Amoxicillin, Azithromycin, Cefpirome and Amikacin all exhibit higher potency than natural products^5–7^. Pleuromutilin (Figure 1, **1**), a diterpenoid natural product from the *basidiomycete* species, shows moderate activities against Gram-positive strains and mycoplasmas^8^^,^^9^. Four semi-synthesised marketed drugs based on its structure have already been developed. Tiamulin and valnemulin (Figure 1, **2** and **3**) can effectively prevent and control swine dysentery, mycoplasmal diseases such as enzootic pneumonia and chronic respiratory disease in poultry^10^^,^^11^. Retapamulin (Figure 1, **4**) is used for the treatment of skin impetigo^12^^,^^13^. Lefamulin (Figure 1, **5**) is approved for treatment of community-acquired bacterial pneumonia (CABP)^14^. These derivatives can inhibit bacterial protein synthesis via specific interaction with 23S rRNA of the 50S bacterial ribosome unit^15^, which are unaffected by resistance to major antibiotic classes, such as beta-lactam antibiotics, tetracyclines, macrolides, fluoroquinolones, and others. Thus, pleuromutilin is a promising candidate for treating drug-resistant bacteria infections^16^, and many compounds derived from four marketed drugs using the thioether as the linkage at the C14 side chain were developed in recent years^17–20^. To increase side chain diversity and improve modification success rate, the bioactive moiety piperazinyl urea, displaying multiple biological activities such as antifungal^21^, analgesic^22^, antibacterial^23^ and antitumour^24^ effects was introduced. In this study, a series of pleuromutilin derivatives **6a**∼**z** with piperazinyl urea (Scheme 1) were efficiently synthesised. Their activities were evaluated against MRSA and Gram-negative strains, and interactions were examined by molecular docking.

## Results and discussion

### Chemistry

**Scheme 2.**
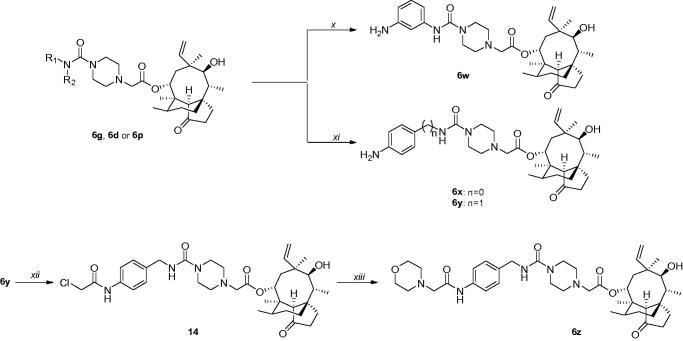
Synthesis route of target compounds **6w ∼ z**. Reagents and conditions: (*x*) TFA, DCM, 25 °C from **6g**; (*xi*) SnCl_2_, EtOH, reflux from **6d** or **6p**; (*xii*) Chloroacetyl chloride, triethylamine, THF, 25 °C; (*xiii*) Morpholine, K_2_CO_3_, THF, reflux.

The piperazinyl urea linkage can be constructed with substituted amines via three strategies (Scheme 1). Pleuromutilin **1** was reacted with tosyl chloride and triethylamine in DCM to form pleuromutilin-22-*O*-tosylate **7** in 78.3% yield, which could convert to 22-(piperazine-1-yl)-22-deoxypleuromutilin **8** by treatment with NaI, piperazine and potassium carbonate in dry THF with a 75.2% yield. Compounds **6a ∼ e** were synthesised by treating **7** with *N*-substituted-1-carboxamide **9a ∼ e** in the presence of a catalytic amount of NaI and K_2_CO_3_ in MeCN with 78.3 ∼ 92.3% yield. Here, the primary amines **12a**∼**e** reacted with bis(trichloromethyl)carbonate (BTC) or *N*, *N'*-carbonyldiimidazole (CDI) to form the corresponding isocyanate, which was used to form piperazinyl urea moiety by reacting with *N*-Boc-piperazine in the presence of triethylamine with 56.2 ∼ 82.3% yield. Afterwards, **9a ∼ e** were obtained via removal of the above Boc protected group using trifluoroacetic acid (TFA) in 93.7 ∼ 97.2% yield. Compounds **6f ∼ u** were synthesised in 45.6 ∼ 82.7% yield by treating **8** in the presence of potassium carbonate with substituted 2,2,2-trichloro-acetamide **10f**∼**u**, which were acquired by reacting primary amines **12f**∼**u** with trichloroacetyl chloride in DCM with 54.7 ∼ 97.2% yield. Moreover, in regard to the secondary amine, the expected product was rarely obtained using the above two strategies. *N*-methylaniline **12v** was treated with 4-nitrophenyl carbonochloridate in the presence of *N*-methylmorpholine (NMM) in DCM to give **11**, which was condensed without further purification and reacted with 22-(piperazine-1-yl)-22-deoxypleuromutilin **8** to form *N*-methyl derivative **6v** in 40.5% yield. Additionally, **6w**∼**z** were synthesised from corresponding Boc-protected amine and nitro molecules (Scheme 2). **6w** was gained from deprotecting **6g** in the presence of TFA in 95.6% yield, and two other amine derivatives **6x** and **6y** were attained by stannous chloride reduction of **6d** and **6p** in 92.5% and 75.5% yields, respectively. **6y** was used to react with chloracetyl chloride to get **14** in the presence of triethylamine with 70.1% yield, which was treated with morpholine to produce **6z** in 50.3% yield.

### Biological activities

MIC and MBC values of the synthesised compounds against three Gram-positive bacteria (i.e.: *Staphylococcus aureus* (*S. aureus*) ATCC 25923, MRSA ATCC 33591 and ATCC 43300) and one Gram-negative bacterium (i.e.: *Escherichia coli* (*E. coli*) ATCC 25922) were determined using the broth microdilution method with the marketed antibiotic Tiamulin as reference. The lipophilicities (Clog*P*) were predicted by ACD/Labs (https://www.acdlabs.com/resources/ilab/).

As Table 1 indicates, almost all the synthesised compounds exhibited broad-spectrum antibacterial activities and showed high potency against both Gram-positive and Gram-negative bacteria with MIC values of 0.5 ∼ 4 μg/mL. Moreover, the majority of synthesised compounds displayed bactericidal activities against MRSA ATCC 33591, as MBC/MIC values were less than 4. Among them, the antibacterial activities of **6d**, **6o**, **6p** and **6q** were at least 2 times stronger than those of Tiamulin. In particular, compound **6p** had a 0.125 μg/mL MIC value (8 times lower than that of Tiamulin) and displayed the best performance against MRSA ATCC 33591. **6p** could also be considered as a bactericide, since MBC/MIC values against four tested strains were less than 2. Furthermore, its activity against *E. coli* ATCC 25922 was 16 times stronger than those of Tiamulin.

**Table 1.** MIC and MBC values of pleuromutilin derivatives possessing piperazinyl urea linkage.

Cpd	MRSA ATCC33591		MRSA ATCC43300		*S. aureus*ATCC25923		*E.coli*ATCC25922		CLog*P*
	MIC	MBC	MIC	MBC	MIC	MBC	MIC	MBC	
6a	1	2	1	>4	1	2	1	>4	4.94
6b	1	2	0.5	2	1	2	1	>4	4.78
6c	0.5	1	0.5	2	1	4	0.5	2	5.28
6d	0.5	1	0.5	2	0.5	2	0.5	2	5.47
6e	1	2	1	>4	1	2	1	4	4.56
6f	2	4	2	4	2	4	4	>16	4.35
6g	1	4	1	4	1	2	1	>4	6.26
6h	1	2	0.5	1	1	1	1	>4	5.33
6i	1	4	1	>4	1	2	4	>16	6.32
6j	4	8	4	16	4	16	32	32	4.42
6k	1	2	0.5	2	1	4	1	>4	3.68
6l	1	2	1	>4	1	4	1	4	5.22
6m	2	4	1	4	0.5	2	1	>4	5.74
6n	0.5	2	1	2	0.5	2	0.5	2	5.19
6o	0.5	2	0.5	>2	0.5	0.5	0.5	2	5.85
6p	0.125	0.125	0.25	0.25	0.125	0.25	0.125	0.125	5.01
6q	0.5	2	0.25	0.25	0.125	0.5	1	>4	5.85
6r	1	4	2	4	1	4	2	8	6.02
6s	1	1	1	2	1	4	1	4	5.93
6t	2	2	2	8	2	4	2	4	5.24
6u	4	8	4	4	4	4	4	8	3.59
6v	2	8	4	16	4	16	4	16	4.94
6w	2	4	1	2	2	8	2	>8	3.85
6x	1	4	1	4	1	>4	1	4	3.25
6y	1	1	1	2	1	>4	1	4	4.00
6z	1	1	1	4	1	4	1	4	4.30
Tiamulin (2)	1	4	1	>4	1	>4	2	>8	5.93

The effects of terminal ring types (e.g. phenyl, pyridinyl, morpholinyl and piperidinyl), ring substituents and distance between rings and urea groups on changes in activities were investigated. MIC value of phenyl piperazinyl urea pleuromutilin **6a** was 1 μg/mL against four strains and when substituents were introduced to the terminal benzene, activities varied within a small range. Compound **6d** with electron-withdrawing 4-nitro groups was the most potent among all phenyl derivatives and exhibited an MIC value of 0.5 μg/mL. However, the introduction of *N*-methyl groups to the urea led to decreased activities, since actions of compound **6v** was 1/4 or 1/2 of compound **6a**. To render the side chain more flexible, a CH_2_ group was introduced to increase the distance between terminal rings and urea groups. As result, the benzyl derivatives with an additional methylene group could noticeably improve activity level. In addition, similar as the effect of substituents on phenyl piperazinyl urea pleuromutilin activity, compound **6p** with the 4-nitro benzyl group exhibited the best performance among all the benzyl derivatives. However, continuously extending the space between the benzene and piperazinyl urea exerts a slight influence on activities. Activity declined when the phenyl group was replaced by 4-pyridnyl group, but phenyl group replacement by substituting 2-pyridinyl, 3-pyridinyl or 5-quinolinyl groups had nearly zero impact on activities. Activity level visibly decreased when aliphatic rings such as piperidine and morpholine were added. The majority of target molecules with an aromatic terminal ring were fairly lipophilic with a CLog*P* value of greater than 5. To enhance hydrophilic properties, compounds **6w**∼**y** with amino groups were produced from corresponding Boc-protected amines and nitro compounds. Their Clog*P* values were approximately equal to or less than 4. Furthermore, a water-soluble morpholine ring was attached to the terminal of **6y** through *N*-acetyl group. Although hydrophilic properties were clearly optimised via the above modification, activity levels were only slightly changed.

Since compound **6p** showed the most potent antibacterial activities against MRSA ATCC 33591 and *E. coli* ATCC 25922, its bactericidal properties were examined by *in vitro* time-kill assay. Bactericidal properties of **6p** against two strains were evaluated at concentrations of 1 × MIC and 6 × MIC, and two concentrations of Tiamulin were set as controls (Figure 2). The MRSA ATCC 33591 propagation-inhibition effect of **6p** was visibly higher at the 1 × MIC concentration than that of the Grown control, which shows similarity to inhibition effects of Tiamulin at 1 × MIC. Meanwhile, at 6 × MIC, compound **6p** achieved complete bactericidal effects in 12 h, a much faster rate than that under 1 × MIC (24 h). In addition, compound **6p** displayed better bactericidal effects against *E. coli* ATCC 25922 compared with Tiamulin at 1 × MIC, and was capable of eliminating total bacteria colony in 24 h, while Tiamulin could only limit bacterial propagation speed. Outcomes were nearly identical at the 6 × MIC concentration.

### Cytotoxicity evaluation

The cytotoxicity of the most potent compound **6p** was investigated in normal human hepatic cell line LO2 and human *embryo* kidney cell line HEK293T using a cell counting kit-8 (CCK-8) assay^25^^,^^26^ (Figure 3). The CCK-8 assay is a colorimetric assay based on the reduction of dye WST-8 [2–(2-methoxy-4-nitrophenyl)-3–(4-nitrophenyl)-5–(2,4disulfophenyl)-2*H*-tetrazolium, monosodium salt] to a water-soluble orange-coloured formazan via dehydrogenase in viable cells. The amount of formazan dye produced by cellular dehydrogenase is correlated with the number of living cells. The result showed that **6p** hardly affected cell viability at concentrations ranging from 0.125 μg/mL to 4 μg/mL in either of the two cell lines. More than 90% of cells remained alive at the 4 μg/mL concentration, indicating **6p** exhibited almost no cytotoxicity even at the concentration of 32 × MIC.

### Docking studies

Docking investigation was conducted to examine the binding mode between synthesised compounds and the peptidyl transferase centre (PTC) of the 50S ribosomal subunit. The method was confirmed by re-docking, revealing that the docking pose of the original ligand Tiamulin nearly coincided with the X-ray structure conformation of RMSD 0.9 Å. All of the title compounds were used for the docking evaluation (Figs. S36–39). Among them, phenyl derivative **6d** and benzyl derivative **6p** with the most potent antibacterial activity, and aliphatic rings derivative **6z** with moderate activity were chosen for the binding mode study. The favourably docked molecules were ranked according to their Grid_Score, which consisted of two parameters: Grid_vdw_energy (mainly referring to hydrophobic interactions and π–π stacking) and Grid_es_energy (often containing hydrogen bonds and salt bridges). The results presented in Table 2 showed that the order of Grid_Scores went in decreasing order (**6p **>** 6d **>** 6z**>Tiamulin), which matched those found in the biological assay.

**Table 2.** Molecular docking analysis of PTC with selected ligands (kcal/mol).

Cpd	Grid_Score	Grid_vdw_energy	Grid_es_energy
6d	−107.27	−103.75	−3.52
6p	−112.91	−111.64	−1.27
6z	−105.50	−98.69	−6.81
Tiamulin	−95.34	−91.14	−4.20

As shown by the superimposed poses in Figures 4(a,b), three synthesised ligands **6d** (green), **6p** (yellow) and **6z** (magenta) were located at the same pocket of PTC as Tiamulin (blue). The tricyclic skeleton of the synthesised compounds was in good agreement with that of Tiamulin, while the side chain at C14 varied noticeably. Many active interactions occurred continuously between the above-mentioned ligands and residues C2565, U2564, G2484, G2044 and A2405.

The important intermolecular PTC-ligand interactions are depicted in Figure 5. In addition to hydrogen bonds between the hydroxyl group on C11 and residue G2484, and between the carbonyl group on C21 and residue G2044, **6d** (Figure 5(a)) and **6p** (Figure 5(b)) were able to interact with A2045 by π-π stacking; the former adopting face-to-face stacking, and the latter displaying nearly edge-to-face stacking. Moreover, the terminal nitro group was able to form one additional hydrogen bond by interacting with C2565 or A2045. **6z** (Figure 5(c)) was able to interact with residues G2484, U2564 and C2565 by three hydrogen bonds, however the π-π stacking was missing. This might explain why the Grid_vdw_energy of **6z** (-98.69 kcal/mol) was worse than **6d** (-103.75 kcal/mol) and **6p** (-111.64 kcal/mol). The hydrophobic environment of the most potent compound **6p** is shown in Figure 5(d). Residues G2484, U2483, C2431, U2485, A2430, U2564, C2565, A2045, A2418, C2420, C2046, G2044 and A2482 were involved in the hydrophobic interactions, which provided strong van der Waals forces in conjunction with the above mentioned π–π stacking of **6p**.

## Experimental

### Synthesis

Analytical grade reagents were used and purchased from Energy Chemical (Shanghai, China) and Kelong Chemical (Chengdu, China). Melting points were determined on an Shenguang WRS-1B apparatus (Shanghai, China). ^1^H NMR and ^13 ^C NMR spectra were measured on a Bruker AV400 spectrometer in CDCl_3_ or DMSO-d_6_. Mass spectra were recorded with a X500R mass spectrometer (AB SCIEX) using the electro spray ionisation (ESI) method.

#### Synthesis of compound 7 and 8

Tosyl chloride (1.91 g, 10.0 mmol) in DCM (10 ml) was added to a solution of pleuromutilin (3.56 g, 9.40 mmol) in DCM (10 ml) containing triethylamine (4.54 g, 12.0 mmol) at 0 °C, then the reaction was stirred at 25 °C for 20 h and the resultant was washed with water (50 ml). The organic phase was collected, dried over Na_2_SO_4_ and evaporated under vacuum to produce yellow oil, which could be purified by recrystallisation in ethanol to obtain white solid **7** (Yield: 78.3%). Spectral data of **7** were identical to those from reports in the literature^27^.

**7** (533 mg, 1.0 mmol) and NaI (30.0 mg, 0.2 mmol) were stirred in dry THF (5 ml) at 25 °C for 0.5 h, then K_2_CO_3_ (276 mg, 2.0 mmol) and piperazine (172 mg, 2.0 mmol) were added to the aforementioned solution. The mixture was heated to reflux and stirred for 6 h. After cooling to room temperature, the resulting mixture was washed with water and condensed until dry. Compound **8** was collected after silica gel column chromatography (Yield: 75.2%). Spectral data results of **8** were identical to those from reports in the literature^28^.

#### Synthesis of compounds 9a ∼ e

Compounds **9a ∼ d** were synthesised according to previous literature^29^^,^^30^, and **9e** was synthesised as follows:

BTC (386 mg, 1.3 mmol) in dry THF (4 ml) was added dropwise to a mixture of triethylamine (526 mg, 5.2 mmol) and **12e** (173 mg, 1.2 mmol) in dry THF (6 ml) solution under an ice-water bath. The mixture was then stirred for 0.5 h. After condensation, *N*-Boc piperazine (503 mg, 2.7 mmol), triethylamine (546 mg, 5.4 mmol) and DCM (10 ml) were added and stirred for 2 h at 25 °C. The solvent was then evaporated before crude product purification via silica gel column chromatography to produce compound **13e**.

Compound **13e** (356 mg, 1.0 mmol) was added to the mixture of 6 ml DCM and TFA (v/v = 10/1) and stirred at 25 °C until **13e** completely converted. The resulting solution was washed with saturated aqueous NaHCO_3_ to neutralise TFA, then f the organic phase was collected and dried over anhydrous Na_2_SO_4_. After filtering, the filtrate was concentrated to acquire the crude product, which was purified via silica gel chromatography to produce compound **9e**.

**13e**: white powder; yield: 56.2%; mp: 222.9–223.8 °C; ^1^H NMR (400 MHz, CDCl_3_): δ (ppm) 8.86 (dd, *J* = 4.0, 1.4 Hz, 1H), 8.18 − 8.05 (m, 1H), 7.92 (d, *J* = 8.4 Hz, 1H), 7.65 − 7.52 (m, 1H), 7.43 (d, *J* = 7.2 Hz, 1H), 7.33 (dd, *J* = 8.4, 4.0 Hz, 1H), 6.89 (s, 1H), 3.45 (s, 8H), 1.48 (s, 9H); ^13 ^C NMR (100 MHz, CDCl_3_): δ (ppm) 156.2, 154.7, 150.4, 148.8, 134.2, 131.0, 129.2, 127.3, 124.4, 122.4, 121.0, 80.5, 28.5; HRMS: calculated for C_19_H_24_N_4_O_3_ ([M + H]^+^): 357.1921, found 357.1929.

**9e**: yellow liquid; yield: 96.3%; ^1^H NMR (400 MHz, DMSO-d_6_): δ (ppm) 9.08 (s, 1H), 8.87 (d, *J* = 4.0 Hz, 1H), 8.35 (d, *J* = 8.4 Hz, 1H), 7.84 (d, *J* = 8.4 Hz, 1H), 7.70 (t, *J* = 8.0 Hz, 1H), 7.54 − 7.44 (m, 2H), 3.80 − 3.72 (m, 4H), 3.24 − 3.17 (m, 4H); ^13 ^C NMR (100 MHz, DMSO-d_6_): δ (ppm) 156.3, 150.7, 148.6, 136.1, 132.9, 129.4, 126.3, 124.8, 123.1, 121.2, 43.1, 41.5; HRMS: calculated for C_14_H_16_N_4_O ([M + H]^+^): 257.1391, found 257.1402.

#### Synthesis of compounds 10f ∼ u

Compounds **10f**, **10h**, **10i**, **10j**, **10l**, **10m**, **10o**, **10r**, **10t** and **10u** were synthesised as in literature^31^^,^^32^. The remaining compounds were synthesised as follows:

A mixture of trichloroacetyl chloride (218 mg, 1.2 mmol) in an appropriate amount of DCM was added to a DCM (3 ml) solution containing compounds **12g** (208 mg, 1.0 mmol) and triethylamine (121 mg, 1.2 mmol) under an ice-water bath. Next, the mixture was stirred at 25 °C until **12g** was completely dispersed. The reaction mixture was evaporated by vacuum and residue was dissolved in DCM. After washing using water and saturated aqueous ammonium chloride, the organic layer was separated and dried over anhydrous Na_2_SO_4_. The crude compound was attained after evaporating the solvent, which was recrystallised by ethanol/water to give **10g**. Compounds **10k**, **10n**, **10p**, **10q** and **10s** were synthesised in the same manner.

**10g**: white powder; yield: 97.2%; mp: 124.9–126.3 °C; ^1^H NMR (400 MHz, CDCl_3_): δ (ppm) 8.33 (s, 1H), 7.78 (t, *J* = 2.0 Hz, 1H), 7.42 − 7.35 (m, 1H), 7.29 (t, *J* = 8.0 Hz, 1H), 7.08 − 7.00 (m, 1H), 6.62 (s, 1H), 1.52 (s, 9H); ^13 ^C NMR (100 MHz, CDCl_3_): δ (ppm) 159.2, 152.7, 139.3, 136.7, 129.8, 115.7, 114.7, 110.2, 92.8, 81.0, 28.3; HRMS: calculated for C_13_H_15_Cl_3_N_2_NaO_3_ ([M + Na]^+^): 375.0040, found 375.0046.

**10k**: white powder; yield: 54.1%; mp: 148.1–149.3 °C; ^1^H NMR (400 MHz, CDCl_3_): δ (ppm) 8.57 (s, 1H), 7.56 − 7.45 (m, 2H), 6.35 (d, *J* = 7.6 Hz, 1H), 4.41 (s, 2H); ^13 ^C NMR (100 MHz, CDCl_3_): δ (ppm) 159.2, 157.5, 148.3, 140.5, 106.1, 103.3, 92.8; HRMS: calculated for C_7_H_6_Cl_3_N_3_O ([M + H]^+^): 253.9649, found 253.9652.

**10n:** white powder; yield: 88.1%; mp: 84.9–85.6 °C; ^1^H NMR (400 MHz, CDCl_3_): δ (ppm) 7.26 − 7.22 (m, 2H), 6.92 − 6.88 (m, 2H), 4.48 (d, *J* = 5.6 Hz, 2H); ^13 ^C NMR (100 MHz, CDCl_3_): δ (ppm) 161.8, 159.6, 129.3, 128.3, 114.4, 92.6, 55.4, 45.0; HRMS: calculated for C_10_H_10_Cl_3_NNaO_2_ ([M + Na]^+^): 303.9669, found 303.9685.

**10p:** light yellow powder; yield: 85.2%; mp: 87.2–88.4 °C; ^1^H NMR (400 MHz, CDCl_3_): δ (ppm) 8.24 (d, *J* = 8.8 Hz, 2H), 7.49 (d, *J* = 8.8 Hz, 2H), 4.67 (d, *J* = 6.0 Hz, 2H); ^13 ^C NMR (100 MHz, CDCl_3_): δ (ppm) 162.4, 147.7, 143.7, 128.3, 124.2, 92.2, 44.5; HRMS: calculated for C_9_H_7_Cl_3_N_2_O_3_ ([M + H]^+^): 296.9595, found 296.9601.

**10q:** white powder; yield: 87.5%; mp: 77.3–78.7 °C; ^1^H NMR (400 MHz, CDCl_3_): δ (ppm) 7.70 (d, *J* = 7.6 Hz, 1H), 7.59 − 7.57 (m, 2H), 7.48 − 7.44 (m, 1H), 4.73 (dd, *J* = 6.0, 0.8 Hz, 2H), 1.25 (s, 1H); ^13 ^C NMR (100 MHz, CDCl_3_): δ (ppm) 162.0, 134.5, 132.7, 130.8, 128.4, 128.3 (q, *J* = 31 Hz), 126.4 (q, *J* = 6 Hz), 124.3 (q, *J* = 272 Hz), 92.4, 42.1; HRMS: calculated for C_10_H_7_Cl_3_F_3_NO ([M + H]^+^): 319.9618, found 319.9624.

**10s:** white powder; yield: 86.8%; mp: 48.1–49.5 °C; ^1^H NMR (400 MHz, CDCl_3_): δ (ppm) 7.26 − 7.25 (m, 1H), 6.84 − 6.83 (m, 2H), 6.78 (t, *J* = 2.0 Hz, 1H), 3.83 (s, 3H), 3.65 (q, *J* = 6.8 Hz, 2H), 2.92 (d, *J* = 6.8 Hz, 2H); ^13 ^C NMR (100 MHz, CDCl_3_): δ (ppm) 161.8, 160.0, 139.4, 129.9, 121.1, 114.4, 112.4, 92.6, 55.2, 42.5, 35.1; HRMS: calculated for C_11_H_12_Cl_3_NO_2_ ([M + H]^+^): 296.0006, found 296.0022.

#### Synthesis of compound 11^33^

4-Nitrophenyl chloroformate (97 mg, 0.48 mmol) and NMM (143 mg, 1.41 mmol) were added to a DCM (5 ml) solution containing N-methylaniline **12v** (51 mg, 0.47 mmol) at 0 °C. The subsequent mixture was stirred for 2 h before pouring into water (5 ml). The organic layer was separated and dried over anhydrous Na_2_SO_4_ After solvent evaporation, the residue was directly used for the next step (Yield: 97.5%).

#### Synthesis of compounds 6a ∼ e

Pleuromutilin 22-tosylate **7** (213 mg, 0.4 mmol) and NaI (14.9 mg, 0.1 mmol) in MeCN (5 ml) were stirred at 25 °C for 0.5 h, then compounds **9a**∼**e** (0.5 mmol) and K_2_CO_3_ (111 mg, 0.8 mmol) were added. The mixture was stirred under 70 °C for 5 h. After solvent evaporation, the crude product was purified via silica gel column chromatography to obtain pure products **6a**∼**e**.

**6a**: white powder; yield: 78.3%; mp: 98.6–100.4 °C; ^1^H NMR (400 MHz, CDCl_3_): δ (ppm) 7.39 − 7.11 (m, 4H), 6.95 (t, *J* = 6.8 Hz, 1H), 6.61 (s, 1H), 6.43 (dd, *J* = 17.2, 11.2 Hz, 1H), 5.73 (d, *J* = 8.0 Hz, 1H), 5.27 (d, *J* = 10.8 Hz, 1H), 5.13 (d, *J* = 17.2 Hz, 1H), 3.47 (s, 4H), 3.29 (s, 1H), 3.11 (ABq, *J* = 17.2 Hz, 2H), 2.70 − 2.38 (m, 4H), 2.36 − 1.92 (m, 5H), 1.81 − 1.26 (m, 11H), 1.10 (s, 3H), 1.07 − 0.98 (m, 1H), 0.82 (d, *J* = 6.8 Hz, 3H), 0.66 (d, *J* = 6.8 Hz, 3H); ^13 ^C NMR (100 MHz, CDCl_3_): δ (ppm) 217.3, 169.0, 155.1, 139.2, 139.1, 128.9, 123.2, 120.2, 117.3, 74.6, 68.6, 59.8, 58.3, 52.6, 45.5, 45.1, 44.0, 43.9, 41.8, 36.8, 36.1, 34.5, 30.5, 26.9, 26.5, 24.9, 16.8, 15.0, 11.6; HRMS: calculated for C_33_H_47_N_3_O_5_ ([M + H]^+^): 566.3588, found 566.3588.

**6b**: white powder; yield: 87.6%; mp: 121.6–122.4 °C; ^1^H NMR (400 MHz, DMSO-d_6_): δ (ppm) 8.33 (s, 1H, NH), 7.33 (d, *J* = 8.8 Hz, 2H), 6.81 (d, *J* = 8.8 Hz, 2H), 6.18 (dd, *J* = 17.2, 11.2 Hz, 1H), 5.60 (d, *J* = 8.0 Hz, 1H), 5.09 − 5.05 (m, 2H), 4.54 (d, *J* = 6.0 Hz, 1H), 3.70 (s, 3H), 3.53 − 3.34 (m, 5H), 3.30 − 3.01 (m, 2H), 2.45 − 2.35 (m, 4H), 2.28 − 2.01 (m, 5H), 1.75 − 1.21 (m, 11H), 1.07 (s, 3H), 1.03 − 0.95 (m, 1H), 0.83 (d, *J* = 6.8 Hz, 3H), 0.64 (d, *J* = 6.8 Hz, 3H); ^13 ^C NMR (100 MHz, DMSO-d_6_): δ (ppm) 217.3, 155.2, 154.4, 141.0, 133.5, 121.5, 115.2, 113.5, 72.6, 68.3, 59.8, 58.9, 57.3, 55.1, 51.8, 45.0, 44.1, 43.7, 41.5, 36.5, 36.4, 34.0, 30.2, 28.6, 26.6, 24.5, 20.8, 16.0, 14.6, 11.6; HRMS: calculated for C_34_H_49_N_3_O_6_ ([M + H]^+^): 596.3694, found 596.3693.

**6c**: white powder; yield: 86.4%; mp: 107.9–109.1 °C; ^1^H NMR (400 MHz, CDCl_3_): δ (ppm) 7.42 − 7.13 (m, 5H), 6.46 (dd, *J* = 17.2, 11.2 Hz, 1H), 5.74 (d, *J* = 8.4 Hz, 1H), 5.29 (d, *J* = 11.2 Hz, 1H), 5.15 (d, *J* = 17.2 Hz, 1H), 4.75 (d, *J* = 5.2 Hz, 1H), 4.37 (d, *J* = 5.2 Hz, 2H), 3.39 (t, *J* = 4.8 Hz, 4H), 3.31 (s, 1H), 3.11 (ABq, *J* = 17.2 Hz, 2H), 2.71 − 2.40 (m, 4H), 2.40 − 1.96 (m, 5H), 1.83 − 1.29 (m, 11H), 1.12 (s, 3H), 1.10 − 1.02 (m, 1H), 0.84 (d, *J* = 6.8 Hz, 3H), 0.67 (d, *J* = 6.8 Hz, 3H); ^13 ^C NMR (100 MHz, CDCl_3_): δ (ppm) 217.2, 169.0, 157.6, 139.4, 139.2, 128.7, 127.9, 127.4, 117.4, 74.7, 68.5, 59.9, 58.3, 52.6, 45.6, 45.1, 44.1, 43.8, 41.9, 36.8, 36.2, 34.6, 30.5, 26.9, 26.5, 24.9, 16.8, 15.0, 11.6; HRMS: calculated for C_34_H_49_N_3_O_5_ ([M + H]^+^): 580.3745, found 580.3741.

**6d**: yellow powder; yield: 88.5%; mp: 87.6–88.9 °C; ^1^H NMR (400 MHz, CDCl_3_): δ (ppm) 8.19 − 8.09 (m, 2H), 7.55 − 7.46 (m, 2H), 6.94 (s, 1H), 6.49 (dd, *J* = 17.2, 11.2 Hz, 1H), 5.79 (d, *J* = 8.4 Hz, 1H), 5.33 (dd, *J* = 11.2, 1.2 Hz, 1H), 5.19 (dd, *J* = 17.2, 1.2 Hz, 1H), 3.57 (t, *J* = 4.8 Hz, 4H), 3.41 − 3.30 (m, 1H), 3.15 (ABq, *J* = 17.2 Hz, 2H), 2.71 − 2.53 (m, 4H), 2.39 − 2.02 (m, 5H), 1.93 − 1.32 (m, 11H), 1.16 (s, 3H), 1.14 − 1.07 (m, 1H), 0.87 (d, *J* = 6.8 Hz, 3H), 0.71 (d, *J* = 6.8 Hz, 3H); ^13 ^C NMR (100 MHz, CDCl_3_): δ (ppm) 217.3, 169.0, 153.8, 145.5, 142.6, 139.2, 125.2, 118.6, 117.4, 74.7, 68.7, 59.7, 58.3, 52.5, 45.6, 45.1, 44.2, 44.1, 41.9, 36.8, 36.2, 34.6, 30.5, 27.0, 26.5, 25.0, 16.9, 15.0, 11.6; HRMS: calculated for C_33_H_46_N_4_O_7_ ([M + H]^+^): 611.3439, found 611.3440.

**6e**: white powder; yield: 92.3%; mp: 138.1–139.3 °C; ^1^H NMR (400 MHz, CDCl_3_): δ (ppm) 8.98 − 8.85 (m, 1H), 8.22 − 812 (m, 1H), 7.96 − 7.89 (m, 1H), 7.69 − 7.60 (m, 1H), 7.57 − 7.48 (m, 1H), 7.43 − 7.34 (m, 1H), 6.70 (d, *J* = 16.0 Hz, 1H), 6.59 − 6.43 (m, 1H), 5.80 (d, *J* = 8.0 Hz, 1H), 5.35 (d, *J* = 11.2 Hz, 1H), 5.21 (d, *J* = 17.2 Hz, 1H), 3.59 (d, *J* = 4.8 Hz, 4H), 3.42 − 3.30 (m, 1H), 3.15 (ABq, *J* = 17.2 Hz, 2H), 2.77 − 2.51 (m, 4H), 2.45 − 2.00 (m, 5H), 1.87 − 1.33 (m, 11H), 1.17 (s, 3H), 1.14 − 1.07 (m, 1H), 0.88 (d, *J* = 5.6 Hz, 3H), 0.73 (d, *J* = 5.6 Hz, 3H); ^13 ^C NMR (100 MHz, CDCl_3_): δ (ppm) 217.2, 169.1, 155.9, 150.4, 148.9, 139.2, 134.2, 130.9, 129.3, 127.2, 122.1, 121.0, 117.4, 74.7, 68.7, 59.9, 58.3, 52.6, 45.6, 45.2, 44.3, 44.1, 41.9, 36.8, 36.2, 34.6, 30.6, 27.0, 26.6, 25.0, 16.9, 15.0, 11.6; HRMS: calculated for C_36_H_48_N_4_O_5_ ([M + H]^+^): 617.3697, found 617.3695.

#### Synthesis of compounds 6f ∼ u

Compounds **10f ∼ u** (1.0 mmol) and K_2_CO_3_ (276 mg, 2.0 mmol) were added to a stirred solution of compound **8** (402 mg, 0.9 mmol) in anhydrous DMF (10 ml) at 25 °C. The mixture was then heated to 80 °C until **8** was fully converted. Afterwards, the mixture was diluted by water and extracted via DCM (20 ml × 3). Subsequently, the combined organic layer was washed with brine and dried over anhydrous MgSO_4_. Solvent evaporation was completed before the crude product was purified by silica gel column chromatography to produce compounds **6f ∼ u.**

**6f**: white powder; yield: 81.5%; mp: 110.9–113.2 °C; ^1^H NMR (400 MHz, CDCl_3_): δ (ppm) 7.65 (s, 1H), 7.59 (s, 1H), 7.20 − 7.03 (m, 3H), 6.65 (s, 1H), 6.50 (dd, *J* = 17.2, 11.2 Hz, 1H), 5.79 (d, *J* = 8.4 Hz, 1H), 5.33 (dd, *J* = 11.2, 1.2 Hz, 1H), 5.20 (dd, *J* = 17.2, 1.6 Hz, 1H), 3.55–3.50 (m, 4H), 3.36 (s, 1H), 3.15 (ABq, *J* = 17.2 Hz, 2H), 2.68 − 2.46 (m, 4H), 2.40 − 2.04 (m, 8H), 1.81 − 1.32 (m, 11H), 1.16 (s, 3H), 1.14 − 1.08 (m, 1H), 0.87 (d, *J* = 6.8 Hz, 3H), 0.72 (d, *J* = 6.8 Hz, 3H); ^13 ^C NMR (100 MHz, CDCl_3_): δ (ppm) 217.2, 169.1, 168.7, 155.1, 139.7, 139.2, 138.6, 129.4, 117.4, 116.1, 114.7, 111.9, 74.7, 68.7, 59.9, 58.3, 52.7, 45.6, 45.2, 44.1, 44.0, 41.9, 36.9, 36.2, 34.6, 30.6, 27.0, 26.6, 25.0, 24.6, 16.9, 15.0, 11.6; HRMS: calculated for C_35_H_50_N_4_O_6_ ([M + H]^+^): 623.3803, found 623.3804.

**6g**: white powder; yield: 77.4%; mp: 109.7–111.2 °C; ^1^H NMR (400 MHz, CDCl_3_): δ (ppm) 7.51 (s, 1H), 7.20 − 7.10 (m, 2H), 6.88 (dd, *J* = 7.6, 1.6 Hz, 1H), 6.63 − 6.36 (m, 3H), 5.79 (d, *J* = 8.4 Hz, 1H), 5.34 (dd, *J* = 11.2, 1.6 Hz, 1H), 5.20 (dd, *J* = 17.2, 1.6 Hz, 1H), 3.51 (t, *J* = 5.0 Hz, 4H), 3.35 (s, 1H), 3.13 (ABq, *J* = 17.2 Hz, 2H), 2.68 − 2.47 (m, 4H), 2.42 − 2.04 (m, 5H), 1.90 − 1.33 (m, 20H), 1.16 (s, 3H), 1.14 − 1.08 (m, 1H), 0.87 (d, *J* = 6.8 Hz, 3H), 0.72 (d, *J* = 6.8 Hz, 3H); ^13 ^C NMR (100 MHz, CDCl_3_): δ (ppm) 217.2, 169.1, 154.8, 152.9, 139.8, 139.2, 139.0, 129.5, 117.5, 114.5, 113.1, 109.8, 80.7, 74.7, 68.6, 60.5, 59.9, 58.3, 52.7, 45.6, 45.2, 44.1, 44.0, 41.9, 36.9, 36.2, 34.6, 30.6, 28.5, 27.0, 26.5, 25.0, 16.9, 15.0, 11.6; HRMS: calculated for C_38_H_56_N_4_O_7_ ([M + H]^+^): 681.4222, found 681.4258.

**6h**: white powder; yield: 82.3%; mp: 103.2–104.4 °C; ^1^H NMR (400 MHz, CDCl_3_): δ (ppm) 7.30 − 7.27 (m, 1H),7.23 − 7.15 (m, 1H), 6.99 (dd, *J* = 8.0, 1.2 Hz, 1H), 6.71 (td, *J* = 8.4, 2.0 Hz, 1H), 6.56 − 6.43 (m, 2H), 5.79 (d, *J* = 8.4 Hz, 1H), 5.34 (dd, *J* = 11.2, 1.2 Hz, 1H), 5.20 (dd, *J* = 17.2, 1.6 Hz, 1H), 3.53 (t, *J* = 5.2 Hz, 4H), 3.35 (d, *J* = 5.6 Hz, 1H), 3.15 (ABq, *J* = 17.2 Hz, 2H), 2.71 − 2.50 (m, 4H), 2.39 − 2.04 (m, 5H), 1.85 − 1.33 (m, 11H), 1.16 (s, 3H), 1.14 − 1.07 (m, 1H), 0.88 (d, *J* = 6.8 Hz, 3H), 0.72 (d, *J* = 6.8 Hz, 3H); ^13 ^C NMR (100 MHz, CDCl_3_): δ (ppm) 217.2, 169.0, 163.2 (*J* = 244 Hz), 154.6, 140.8, 140.7, 139.2, 130.0 (*J* = 9.4 Hz), 117.4, 115.0 (*J* = 3.2 Hz), 114.9, 109.8 (*J* = 21 Hz), 107.4, 107.1, 74.7, 68.7, 60.5, 59.8, 58.3, 52.6, 45.6, 44.1, 41.9, 36.8, 36.2, 34.6, 30.6, 27.0, 26.5, 25.0, 16.9, 15.0, 11.6; HRMS: calculated for C_33_H_46_FN_3_O_5_ ([M + H]^+^): 584.3494, found 584.3488.

**6i**: white powder; yield: 81.5%; mp: 116.3–117.7 °C; ^1^H NMR (400 MHz, CDCl_3_): δ (ppm) 7.62 (s, 1H), 7.55 (d, *J* = 8.0 Hz, 1H), 7.37 (t, *J* = 8.0 Hz, 1H), 7.30 − 7.26 (m, 1H), 6.61 (s, 1H), 6.50 (dd, *J* = 17.2, 11.2 Hz, 1H), 5.79 (d, *J* = 8.4 Hz, 1H), 5.34 (dd, *J* = 11.2, 1.2 Hz, 1H), 5.20 (dd, *J* = 17.2, 1.6 Hz, 1H), 3.55 (t, *J* = 5.2 Hz, 4H), 3.35 (s, 1H), 3.27 (ABq, *J* = 17.2 Hz, 2H), 2.73 − 2.51 (m, 4H), 2.39 − 2.04 (m, 5H), 1.83 − 1.33 (m, 11H), 1.16 (s, 3H), 1.14 − 1.08 (m, 1H), 0.88 (d, *J* = 6.8 Hz, 3H), 0.72 (d, *J* = 6.8 Hz, 3H); ^13 ^C NMR (100 MHz, CDCl_3_): δ (ppm) 217.2, 169.0, 154.6, 139.7, 139.2, 131.5, 131.2, 129.5, 123.0, 119.8, 117.4, 116.6, 74.7, 68.7, 60.5, 59.8, 58.3, 52.6, 45.6, 45.2, 44.1, 41.9, 36.9, 36.2, 34.6, 30.6, 27.0, 26.5, 25.0, 16.9, 15.0, 11.6; HRMS: calculated for C_34_H_46_F_3_N_3_O_5_ ([M + H]^+^): 634.3462, found 634.3468.

**6j**: white powder; yield: 73.2%; mp: 127.9–129.5 °C; ^1^H NMR (400 MHz, CDCl_3_): δ (ppm) 8.33 (d, J = 6.6 Hz, 2H), 7.66 (d, J = 6.2 Hz, 2H), 6.50 (dd, J = 17.2, 11.2 Hz, 1H), 5.80 (d, J = 8.4 Hz, 1H), 5.34 (dd, J = 11.2, 1.6 Hz, 1H), 5.20 (dd, J = 17.2, 1.6 Hz, 1H), 3.64 (t, J = 4.8 Hz, 4H), 3.36 (d, J = 6.6 Hz, 1H), 3.16 (ABq, *J* = 17.2 Hz, 2H), 2.72 − 2.54 (m, 4H), 2.41 − 2.05 (m, 5H), 1.83 − 1.33 (m, 11H), 1.17 (s, 3H), 1.15 − 1.08 (m, 1H), 0.88 (d, J = 6.8 Hz, 3H), 0.72 (d, J = 6.8 Hz, 3H); ^13 ^C NMR (100 MHz, CDCl3): δ (ppm) 217.2, 169.0, 153.8, 150.4, 146.7, 139.2, 117.4, 113.4, 74.7, 68.7, 59.8, 58.3, 52.5, 45.6, 45.2, 44.2, 44.1, 41.9, 36.8, 36.2, 34.6, 30.6, 27.0, 26.6, 25.0, 16.9, 15.0, 11.6; HRMS: calculated for C_32_H_46_N_4_O_5_ ([M + H]^+^): 567.3541, found 567.3546.

**6k**: white powder; yield: 67.8%; mp: 129.6–130.9 °C; ^1^H NMR (400 MHz, CDCl_3_): δ (ppm) 7.43 − 7.35 (m, 1H), 7.31 (d, *J* = 8.0 Hz, 1H), 6.96 (s, 1H), 6.49 (dd, *J* = 17.2, 11.2 Hz, 1H), 6.14 (d, *J* = 7.6 Hz, 1H), 5.78 (d, *J* = 8.4 Hz, 1H), 5.33 (d, *J* = 11.2 Hz, 1H), 5.19 (dd, *J* = 17.2, 1.2 Hz, 1H), 4.25 (s, 2H), 3.54 (t, *J* = 4.8 Hz, 4H), 3.42 − 3.29 (m, 1H), 3.12 (ABq, *J* = 17.2 Hz, 2H), 2.72 − 2.39 (m, 4H), 2.39 − 2.01 (m, 5H), 1.82 − 1.31 (m, 11H), 1.15 (s, 3H), 1.13 − 1.06 (m, 1H), 0.86 (d, *J* = 6.8 Hz, 3H), 0.70 (d, *J* = 6.8 Hz, 3H); ^13 ^C NMR (100 MHz, CDCl_3_): δ (ppm) 217.3, 169.0, 157.0, 153.9, 151.2, 140.2, 139.1, 117.5, 102.9, 102.5, 74.7, 68.6, 59.8, 58.3, 52.6, 45.6, 45.1, 44.1, 43.9, 41.9, 36.8, 36.2, 34.6, 30.5, 26.9, 26.5, 25.0, 16.9, 15.0, 11.6; HRMS: calculated for C_32_H_47_N_5_O_5_ ([M + H]^+^): 582.3650, found 582.3658.

**6l:** white powder; yield: 77.2%; mp: 149.9–151.8 °C; ^1^H NMR (400 MHz, CDCl_3_): δ (ppm) 8.20 (d, *J* = 2.4 Hz, 1H), 7.88 (dd, *J* = 8.8, 2.8 Hz, 1H), 7.38 (d, *J* = 8.8 Hz, 1H), 6.91 (s, 1H), 6.50 (dd, *J* = 17.2, 11.2 Hz, 1H), 5.80 (d, *J* = 8.4 Hz, 1H), 5.34 (dd, *J* = 11.2, 1.2 Hz, 1H), 5.21 (dd, *J* = 17.2, 1.2 Hz, 1H), 3.56 (t, *J* = 4.8 Hz, 4H), 3.45 − 3.31 (m, 1H), 3.15 (ABq, *J* = 17.2 Hz, 2H), 2.69 − 2.51 (m, 4H), 2.40 − 2.06 (m, 5H), 1.84 − 1.34 (m, 11H), 1.17 (s, 3H), 1.15 − 1.07 (m, 1H), 0.89 (d, *J* = 6.8 Hz, 3H), 0.72 (d, *J* = 6.8 Hz, 3H); ^13 ^C NMR (100 MHz, CDCl_3_): δ (ppm) 217.3, 169.0, 154.4, 141.1, 139.2, 135.9, 134.6, 130.3, 127.9, 117.4, 74.7, 68.7, 59.8, 58.3, 52.5, 45.6, 45.1, 44.1, 41.9, 36.8, 36.2, 34.6, 30.5, 26.9, 26.5, 24.9, 16.9, 15.0, 11.6; HRMS: calculated for C_32_H_46_BrN_4_O_5_ ([M + H]^+^): 645.2646, found 645.2648, 647.2638.

**6m**: white powder; yield: 56.2%; mp: 102.3–103.7 °C; ^1^H NMR (400 MHz, CDCl_3_): δ (ppm) 7.19 (d, *J* = 8.0 Hz, 2H), 7.13 (d, *J* = 8.0 Hz, 2H), 6.50 (dd, *J* = 17.2, 11.2 Hz, 1H), 5.79 (d, *J* = 8.4 Hz, 1H), 5.33 (dd, *J* = 11.2, 1.2 Hz, 1H), 5.19 (dd, *J* = 17.2, 1.2 Hz, 1H), 4.63 (t, *J* = 5.2 Hz, 1H), 4.37 (d, *J* = 5.2 Hz, 2H), 3.47 − 3.31 (m, 5H), 3.13 (ABq, *J* = 17.2 Hz, 2H), 2.57–2.50 (m, 4H), 2.33 (s, 3H), 2.25 − 2.04 (m, 5H), 1.80 − 1.74 (m, 1H), 1.70 − 1.42 (m, 10H), 1.16 (s, 3H), 1.13 − 1.08 (m, 1H), 0.87 (d, J = 6.8 Hz, 3H), 0.71 (d, J = 6.8 Hz, 3H); ^13 ^C NMR (100 MHz, CDCl_3_): δ (ppm) 217.2, 169.0, 157.4, 139.0, 137.1, 136.3, 129.3, 127.9, 117.4, 74.6, 68.4, 59.9, 58.2, 52.6, 45.5, 45.0, 44.8, 44.0, 43.6, 41.8, 36.7, 36.1, 34.5, 30.4, 26.8, 26.4, 24.9, 21.1, 16.8, 14.9, 11.6; HRMS: calculated for C_35_H_51_N_3_O_5_ ([M + H]^+^): 594.3901, found 594.3904.

**6n**: white powder; yield: 60.1%; mp: 104.2–105.8 °C; ^1^H NMR (400 MHz, CDCl_3_): δ (ppm) 7.22 (d, *J* = 8.4 Hz, 2H), 6.85 (d, *J* = 8.4 Hz, 2H), 6.49 (dd, *J* = 17.2, 11.2 Hz, 1H), 5.78 (d, *J* = 8.4 Hz, 1H), 5.33 (d, *J* = 11.2 Hz, 1H), 5.19 (d, *J* = 17.2 Hz, 1H), 4.61 (t, *J* = 5.6 Hz, 1H), 4.34 (d, *J* = 5.2 Hz, 2H), 3.79 (s, 3H), 3.45 − 3.41 (m, 4H), 3.35 (dd, *J* = 10.4, 6.4 Hz, 1H), 3.15 (ABq, *J* = 17.2 Hz, 2H), 2.62 − 2.46 (m, 4H), 2.36 − 2.03 (m, 5H), 1.81 − 1.40 (m, 11H), 1.16 (s, 3H), 1.13 − 1.08 (m, 1H), 0.87 (d, *J* = 6.8 Hz, 3H), 0.71 (d, *J* = 6.8 Hz, 3H); ^13 ^C NMR (100 MHz, CDCl_3_): δ (ppm) 217.2, 169.0, 157.4, 139.0, 131.4, 129.2, 117.3, 114.0, 74.6, 68.4, 59.9, 58.2, 55.3, 45.5, 45.0, 44.5, 44.0, 43.6, 41.8, 36.7, 36.1, 34.5, 30.4, 26.8, 26.4, 24.9, 16.8, 14.9, 11.5; HRMS: calculated for C_35_H_51_N_3_O_6_ ([M + H]^+^): 610.3851, found 610.3831.

**6o**: white powder; yield: 53.1%; mp: 109.6–110.7 °C; ^1^H NMR (400 MHz, CDCl_3_): δ (ppm) 7.58 (d, *J* = 8.4 Hz, 2H), 7.41 (d, *J* = 8.4 Hz, 2H), 6.50 (dd, *J* = 17.2, 11.2 Hz, 1H), 5.79 (d, *J* = 8.4 Hz, 1H), 5.34 (dd, *J* = 11.2, 1.2 Hz, 1H), 5.20 (d, *J* = 17.2 Hz, 1H), 4.83 (t, *J* = 5.6 Hz, 1H), 4.48 (d, *J* = 5.6 Hz, 2H), 3.48(t, *J* = 4.4 Hz, 4H), 3.35 (d, *J* = 6.2 Hz, 1H), 3.25 (ABq, *J* = 17.2 Hz, 2H), 2.67 − 2.45 (m, 4H), 2.38 − 2.03 (m, 5H), 1.81 − 1.37 (m, 11H), 1.16 (s, 3H), 1.14 − 1.09 (m, 1H), 0.88 (d, *J* = 6.4 Hz, 3H), 0.72 (d, *J* = 6.8 Hz, 3H); ^13 ^C NMR (100 MHz, CDCl_3_): δ (ppm) 217.1, 168.3, 157.4, 143.6, 139.1, 129.7 (q, *J* = 33 Hz), 127.9, 125.6 (q, *J* = 4 Hz), 117.5, 74.7, 69.0, 59.3, 58.3, 52.7, 45.6, 45.1, 44.6, 44.1, 43.5, 41.9, 36.8, 36.2, 34.6, 30.5, 26.9, 26.5, 25.0, 16.9, 15.0, 11.6; HRMS: calculated for C_35_H_48_F_3_N_3_O_5_ ([M + H]^+^): 648.3619, found 648.3597.

**6p**: light yellow powder; yield: 55.1%; mp: 115.2–116.5 °C; ^1^H NMR (400 MHz, CDCl_3_): δ (ppm) 8.14 (d, *J* = 8.4 Hz, 2H), 7.44 (d, *J* = 8.4 Hz, 2H), 6.49 (dd, *J* = 17.2, 11.2 Hz, 1H), 5.79 (d, *J* = 8.4 Hz, 1H), 5.33 (d, *J* = 11.2 Hz, 1H), 5.21–5.15 (m, 2H), 4.50 (d, *J* = 5.6 Hz, 2H), 3.46 (t, *J* = 4.4 Hz, 4H), 3.36 (s, 1H), 3.15 (ABq, *J* = 17.2 Hz, 2H), 2.62–2.50 (m, 4H), 2.38 − 2.05 (m, 5H), 1.82 − 1.41 (m, 11H), 1.16 (s, 3H), 1.11 (d, *J* = 13.6 Hz, 1H), 0.88 (d, *J* = 6.8 Hz, 3H), 0.71 (d, *J* = 6.8 Hz, 3H); ^13 ^C NMR (100 MHz, CDCl_3_): δ (ppm) 217.1, 168.9, 157.3, 147.4, 147.1, 139.1, 128.0, 123.8, 117.2, 74.6, 68.5, 60.41, 59.7, 58.2, 52.4, 45.5, 45.0, 44.2, 44.0, 43.7, 41.8, 36.7, 36.1, 34.5, 30.4, 26.8, 26.5, 24.8, 16.7, 14.9, 11.5; HRMS: calculated for C_34_H_48_N_4_O_7_ ([M + H]^+^): 625.3596, found 625.3596.

**6q**: white powder; yield: 45.6%; mp: 107.6–108.7 °C; ^1^H NMR (400 MHz, CDCl_3_): δ (ppm) 7.61 (d, *J* = 7.6 Hz, 1H), 7.57 (d, *J* = 7.6 Hz, 1H), 7.49 (t, *J* = 7.6 Hz, 1H), 7.34 (t, *J* = 7.6 Hz, 1H), 6.47 (dd, *J* = 17.2, 11.2 Hz, 1H), 5.76 (d, *J* = 8.4 Hz, 1H), 5.31 (d, *J* = 11.2 Hz, 1H), 5.17 (d, *J* = 17.2 Hz, 1H), 4.93 (d, *J* = 5.6 Hz, 1H), 4.57 (d, *J* = 5.6 Hz, 2H), 3.40 (t, *J* = 4.8 Hz, 4H), 3.33 (dd, *J* = 10.4, 6.8 Hz, 1H), 3.11 (ABq, *J* = 17.2 Hz, 2H), 2.58 − 2.44 (m, 4H), 2.35 − 2.04 (m, 5H), 1.80 − 1.39 (m, 11H), 1.14 (s, 3H), 1.12 − 1.06 (m, 1H), 0.86 (d, *J* = 6.8 Hz, 3H), 0.69 (d, *J* = 6.8 Hz, 3H); ^13 ^C NMR (100 MHz, CDCl_3_): δ (ppm) 217.1, 168.9, 157.2, 139.1, 137.9, 132.3, 130.6, 127.4, 125.9, 125.9, 117.3, 74.6, 68.5, 59.8, 58.2, 52.5, 45.5, 45.0, 44.0, 43.6, 41.8, 41.4, 36.7, 36.1, 34.5, 30.4, 26.8, 26.4, 24.8, 16.7, 14.9, 11.5; HRMS: calculated for C_35_H_48_F_3_N_3_O_5_ ([M + H]^+^): 648.3619, found 648.3620.

**6r**: white powder; yield: 65.1%; mp: 136.5–137.9 °C; ^1^H NMR (400 MHz, CDCl_3_): δ (ppm) 7.32 − 7.27 (m, 2H), 7.24 − 7.15 (m, 3H), 6.49 (dd, *J* = 17.2, 11.2 Hz), 5.78 (d, *J* = 8.4 Hz), 5.33 (dd, J = 11.2, 1.2 Hz), 5.19 (dd, *J* = 17.2, 1.2 Hz), 4.44 (t, 1H, *J* = 5.6 Hz), 3.47 (dd, *J* = 12.4, 6.8 Hz, 2H), 3.35 (t, *J* = 4.8 Hz, 4H), 3.10 (ABq, *J* = 17.2 Hz, 2H), 2.81 (t, *J* = 6.8 Hz, 2H), 2.58 − 2.42 (m, 4H), 2.37 − 2.02 (m, 5H), 1.79 − 1.24 (m, 11H), 1.15 (s, 3H), 1.13 − 1.07 (m, 1H), 0.87 (d, J = 6.4 Hz, 3H), 0.70 (d, *J* = 6.8 Hz, 3H); ^13 ^C NMR (100 MHz, CDCl_3_): δ (ppm) 217.3, 169.0, 157.6, 139.1, 139.5, 129.0, 128.7, 126.5, 117.5, 74.7, 68.5, 59.9, 58.3, 52.6, 45.6, 45.1, 44.1, 43.6, 42.1, 41.9, 36.8, 36.4, 36.2, 34.6, 30.5, 26.9, 26.5, 25.0, 16.9, 15.0, 11.6; HRMS: calculated for C_35_H_51_N_3_O_5_ ([M + H]^+^): 594.3901[M + H]^+^, found 594.3905.

**6s**: white powder; yield: 61.7%; mp: 105.8–107.1 °C; ^1^H NMR (400 MHz, CDCl_3_): δ (ppm) 7.22 (t, *J* = 7.6 Hz, 1H), 6.78 − 6.73 (m, 3H), 6.50 (dd, *J* = 17.2, 11.2 Hz, 1H), 5.78 (d, *J* = 8.4 Hz, 1H), 5.33 (d, *J* = 11.2 Hz, 1H), 5.20 (d, *J* = 17.2 Hz, 1H), 4.42 (t, *J* = 5.6 Hz, 1H), 3.79 (s, 3H), 3.47 (dd, *J* = 12.4, 6.8 Hz, 2H), 3.36 (t, *J* = 4.8 Hz, 4H), 3.12 (ABq, *J* = 17.2 Hz, 2H), 2.79 (t, *J* = 6.8 Hz, 2H), 2.54 − 2.49 (m, 4H), 2.37 − 2.03 (m, 5H), 1.80 − 1.42 (m, 11H), 1.16 (s, 3H), 1.13 − 1.09 (m, 1H), 0.87 (d, *J* = 6.8 Hz, 3H), 0.71 (d, *J* = 6.8 Hz, 3H); ^13 ^C NMR (100 MHz, CDCl_3_): δ (ppm) 217.3, 169.1, 159.9, 157.6, 139.1, 141.1, 129.7, 121.3, 114.6, 111.9, 117.5, 74.7, 68.5, 59.9, 58.3, 55.3, 52.6, 45.6, 45.1, 44.1, 43.6, 42.0, 41.9, 36.8, 36.5, 36.2, 34.6, 30.5, 26.9, 26.5, 25.0, 16.9, 15.0, 11.6; HRMS: calculated for C_36_H_53_N_3_O_6_ ([M + H]^+^): 624.4007, found 624.3995.

**6t**: white powder; yield: 82.7%; mp: 119.5–122.2 °C; ^1^H NMR (400 MHz, CDCl_3_): δ (ppm) 6.49 (dd, *J* = 17.2, 11.2 Hz, 1H), 5.77 (d, *J* = 8.4 Hz, 1H), 5.35 (s, 1H), 5.32 (dd, *J* = 11.2, 1.6 Hz, 1H), 5.18 (dd, *J* = 17.2, 1.6 Hz, 1H), 3.40 (t, *J* = 4.8 Hz, 4H), 3.36 − 3.24 (m, 3H), 3.12 (ABq, *J* = 17.2 Hz, 2H), 2.63 − 1.99 (m, 16H), 1.83 − 1.31 (m, 17H), 1.15 (s, 3H), 1.13 − 1.03 (m, 1H), 0.86 (d, *J* = 6.8 Hz, 3H), 0.70 (d, *J* = 6.8 Hz, 3H); ^13 ^C NMR (100 MHz, CDCl_3_): δ (ppm) 217.3, 169.1, 162.6, 157.9, 139.2, 117.4, 77.5, 77.2, 76.8, 74.7, 68.5, 60.0, 58.3, 57.6, 54.3, 52.7, 45.6, 45.1, 44.0, 43.6, 41.9, 37.3, 36.8, 36.6, 36.2, 34.6, 31.5, 30.5, 26.9, 26.5, 26.0, 25.0, 24.4, 16.8, 15.0, 11.6; HRMS: calculated for C_34_H_56_N_4_O_5_ ([M + H]^+^): 601.4323, found 601.4326.

**6u**: white powder; yield: 79.2%; mp: 92.1–93.8 °C; ^1^H NMR (400 MHz, CDCl_3_): δ (ppm) 7.98 (s, 1H), 6.47 (dd, *J* = 17.2, 11.2 Hz, 1H), 5.76 (d, *J* = 8.4 Hz, 1H), 5.30 (d, *J* = 11.2 Hz, 1H), 5.22 − 5.07 (m, 2H), 3.78 − 3.62 (m, 4H), 3.49 − 3.24 (m, 6H), 3.10 (ABq, *J* = 17.2 Hz, 2H), 2.63 − 2.37 (m, 10H), 2.36 − 1.96 (m, 5H), 1.81 − 1.29 (m, 11H), 1.13 (s, 3H), 1.11 − 1.04 (m, 1H), 0.85 (d, *J* = 6.8 Hz, 3H), 0.69 (d, *J* = 6.8 Hz, 3H); ^13 ^C NMR (100 MHz, CDCl_3_): δ (ppm) 217.2, 169.0, 157.8, 139.2, 117.3, 74.6, 68.5, 67.0, 59.9, 58.3, 57.6, 53.4, 52.6, 45.5, 45.1, 44.0, 43.6, 41.9, 37.0, 36.1, 34.5, 30.5, 26.9, 26.5, 24.9, 16.8, 15.0, 11.6; HRMS: calculated for C_33_H_54_N_4_O_6_ ([M + H]^+^): 603.4116, found 603.4144.

#### Synthesis of compound 6v

The solution of **11** (128 mg, 0.47 mmol) in acetonitrile (10 ml) was treated with 22-(piperazine-1-yl)-22-deoxypleuromutilin **8** (210 mg, 0.47 mmol) and DMAP (57 mg, 0.47 mmol), which was heated to reflux for 24 h. After solvent removal, the residue was dissolved in EtOAc (10 ml). The organic phase was washed with 1 N NaOH and brine, followed by drying over anhydrous Na_2_SO_4_. The filtrate obtained via filtration was concentrated until dry, and then purification by silica gel chromatography was conducted to obtain compound **6v**. white powder; yield: 40.5%; mp: 105.1–106.3 °C; ^1^H NMR (400 MHz, CDCl_3_): δ (ppm) 7.33 − 7.31 (m, 2H), 7.11 − 7.06 (m, 3H), 6.47 (dd, *J* = 17.2, 11.2 Hz, 1H), 5.74 (d, *J* = 8.4 Hz, 1H), 5.30 (dd, *J* = 11.2, 1.2 Hz, 1H), 5.17(dd, *J* = 17.2, 1.2 Hz, 1H), 3.33 (dd, *J* = 10.4, 6.8 Hz, 1H), 3.25 (t, *J* = 4.9 Hz, 4H), 3.21 (s, 3H), 3.02 (ABq, *J* = 17.2 Hz, 2H), 2.40 − 2.23 (m, 7H), 2.26 − 1.99 (m, 2H), 1.80 − 1.38 (m, 11H), 1.14 (s, 3H), 1.12 − 1.05 (m, 1H), 0.85 (d, *J* = 7.2 Hz, 3H), 0.66 (d, *J* = 7.2 Hz, 3H); ^13 ^C NMR (100 MHz, CDCl_3_): δ (ppm) 217.2, 168.9, 160.9, 146.7, 139.0, 129.5, 124.6, 123.8, 117.3, 74.6, 68.3, 59.8, 58.2, 52.5, 45.5, 45.4, 44.9, 43.9, 41.8, 39.6, 36.7, 36.0, 34.5, 30.4, 26.8, 26.4, 24.9, 16.7, 14.9, 11.5; HRMS: calculated for C_34_H_49_N_3_O_5_ ([M + H]^+^): 580.3745, found 580.3744.

#### Synthesis of compound 6w

Compound **6g** (136 mg, 0.2 mmol) was added to a mixture of 6 ml DCM and TFA (v/v = 10/1) and stirred at 25 °C until completely converted. After neutralisation by saturated aqueous NaHCO_3_, the organic phase was collected and dried over anhydrous Na_2_SO_4_. Then, the filtrate obtained via filtration was concentrated to generate the crude product, which was purified by silica gel chromatography to produce compound **6w**. white powder; yield: 95.6%; mp: 146.2–147.5 °C; ^1^H NMR (400 MHz, CDCl_3_): δ (ppm) 7.02 (t, *J* = 8.0 Hz, 1H), 6.95 (t, *J* = 2.0 Hz, 1H), 6.56 − 6.44 (m, 2H), 6.38 − 6.31 (m, 2H), 5.79 (d, *J* = 8.4 Hz, 1H), 5.34 (dd, *J* = 11.2, 1.6 Hz, 1H), 5.20 (dd, *J* = 17.2, 1.2 Hz, 1H), 3.52 (t, *J* = 4.8 Hz, 4H), 3.35 (d, *J* = 6.0 Hz, 1H), 3.12 (ABq, *J* = 17.2 Hz, 2H), 2.68 − 2.49 (m, 4H), 2.41 − 2.02 (m, 7H), 1.86 − 1.21 (m, 13H), 1.16 (s, 3H), 1.14 − 1.07 (m, 1H), 0.87 (d, *J* = 6.8 Hz, 4H), 0.72 (d, *J* = 6.8 Hz, 3H); ^13 ^C NMR (100 MHz, CDCl_3_): δ (ppm) 217.3, 169.1, 155.0, 147.3, 140.0, 139.2, 129.7, 117.5, 110.2, 109.9, 106.8, 74.7, 68.6, 59.9, 58.3, 52.7, 45.6, 45.2, 44.1, 44.0, 41.9, 36.8, 36.2, 34.6, 30.6, 27.0, 26.5, 25.0, 16.9, 15.0, 11.6; HRMS: calculated for C_33_H_48_N_4_O_5_ ([M + H]^+^): 581.3697, found 581.3704.

#### Synthesis of compound 6x and 6y

A mixture of **6d** (122 mg, 0.2 mmol), SnCl_2_ (474 mg, 2.5 mmol) and ethanol (5 ml) was stirred under reflux for 5 h. After cooling to 25 °C, 1 N NaOH was added. The resultant mixture was extracted with DCM (10 ml × 3) and separated. After the organic phase was dried over Na_2_SO_4_ and concentrated, the residue was purified via silica gel column chromatography to acquire **6x**. **6y** was synthesised by a similar method used for **6p**.

**6x**: yellow powder; yield: 92.5%; mp: 137.4–138.7 °C; ^1^H NMR (400 MHz, CDCl_3_): δ (ppm) 7.08 (d, *J* = 8.4 Hz, 2H), 6.62 (d, *J* = 8.4 Hz, 2H), 6.50 (dd, *J* = 17.2, 11.2 Hz, 1H), 6.18 (s, 1H), 5.79 (d, *J* = 8.4 Hz, 1H), 5.34 (d, *J* = 11.2 Hz, 1H), 5.20 (d, *J* = 17.2 Hz, 1H), 3.50 (t, *J* = 4.8 Hz, 4H), 3.35 (d, *J* = 5.2 Hz, 1H), 3.14 (ABq, *J* = 17.2 Hz, 2H), 2.67 − 2.49 (m, 4H), 2.41 − 2.01 (m, 5H), 1.83 − 1.32 (m, 13H), 1.16 (s, 3H), 1.14 − 1.07 (m, 1H), 0.87 (d, *J* = 6.8 Hz, 3H), 0.72 (d, *J* = 6.8 Hz, 3H); ^13 ^C NMR (100 MHz, CDCl_3_): δ (ppm) 217.3, 169.1, 155.8, 142.9, 139.2, 130.2, 123.1, 117.5, 115.7, 74.7, 68.6, 59.9, 58.3, 52.7, 45.6, 45.2, 44.1, 44.0, 41.9, 36.8, 36.2, 34.6, 30.6, 29.8, 27.0, 26.5, 25.0, 16.9, 15.0, 11.6; HRMS: calculated for C_33_H_48_N_4_O_5_ ([M + H]^+^): 581.3697, found 581.3705.

**6y**: light yellow powder; yield: 75.5%; mp: 118.5–119.3 °C; ^1^H NMR (400 MHz, CDCl_3_): δ (ppm) 7.08 (d, *J* = 8.4 Hz, 2H), 6.65 − 6.60 (m, 2H), 6.48 (dd, *J* = 17.2, 11.2 Hz, 1H), 5.77 (d, *J* = 8.4 Hz, 1H), 5.32 (dd, *J* = 11.2, 1.2 Hz, 1H), 5.18 (dd, *J* = 17.2, 1.2 Hz, 1H), 4.59 (t, *J* = 5.6 Hz, 1H), 4.27 (d, *J* = 5.2 Hz, 2H), 3.40 (t, *J* = 5.2 Hz, 4H), 3.35 (s, 1H), 3.11 (ABq, *J* = 17.2 Hz, 2H), 2.56 − 2.46 (m, 4H), 2.36 − 2.02 (m, 6H), 1.74– 1.27 (m, 10H), 1.15 (s, 3H), 1.12 − 1.06 (m, 1H), 0.86 (d, *J* = 6.8 Hz, 3H), 0.70 (d, *J* = 6.8 Hz, 3H); ^13 ^C NMR (100 MHz, CDCl_3_) δ (ppm) 217.1, 168.9, 157.4, 145.8, 139.1, 129.2, 129.1, 117.3, 115.2, 74.6, 68.4, 59.9, 58.2, 52.6, 45.5, 45.0, 44.7, 44.0, 43.6, 41.8, 36.7, 36.1, 34.5, 30.4, 26.8, 26.4, 24.9, 16.7, 14.9, 11.5; HRMS: calculated for C_34_H_50_N_4_O_5_ ([M + H]^+^): 595.3854, found 595.3860.

#### Synthesis of compound 6z

**6y** (190 mg, 0.32 mmol) and triethylamine (49 mg, 0.48 mmol) were dissolved in dry THF, and chloroacetyl chloride (54 mg, 0.48 mmol) was added under an ice bath condition to the above solution. The mixture was stirred at 25 °C for about 4 h before water was added. The resulting mixture was extracted via DCM, washed with brine, and dried over anhydrous Na_2_SO_4_. Then, the organic phase was concentrated to obtain the crude intermediate, which was purified by silica gel column chromatography to produce **14**.

Compound **14** (201 mg, 0.30 mmol), morpholine (52 mg, 0.60 mmol), and K_2_CO_3_ (82 mg, 0.60 mmol) were dissolved in dry THF (5 ml) and maintained at reflux for about 6 h. After the solution was concentrated, the residue was purified by silica gel column chromatography to generate **6z**.

**14**: white powder; yield: 70.1%; mp: 118.1–119.2 °C; ^1^H NMR (400 MHz, CDCl_3_): δ (ppm) 8.25 (s, 1H), 7.49 (d, J = 8.4 Hz, 2H), 7.29 (d, J = 8.4 Hz, 2H), 6.49 (dd, J = 17.2, 11.2 Hz, 1H), 5.79 (d, J = 8.4 Hz, 1H), 5.34 (d, J = 11.2 Hz, 1H), 5.20 (d, J = 17.2 Hz, 1H), 4.77 (s, 1H), 4.39 (d, J = 5.6 Hz, 2H), 4.18 (s, 2H), 3.48 (s, 4H), 3.39 − 3.32 (m, 1H), 3.30 − 3.07 (m, 2H), 2.64 (br, 4H), 2.38 − 2.15 (m, 3H), 2.08 (dd, J = 16.4, 8.0 Hz, 2H), 1.80 − 1.47 (m, 11H), 1.16 (s, 3H), 1.13 − 1.09 (m, 1H), 0.88 (d, J = 6.8 Hz, 3H), 0.71 (d, J = 6.8 Hz, 3H); ^13 ^C NMR (100 MHz, CDCl_3_): δ (ppm) 216.7, 188.9, 164.0, 157.4, 139.0, 135.8, 128.6, 120.6, 117.4, 74.6, 58.2, 52.3, 45.5, 45.0, 44.5, 44.0, 42.9, 41.8, 36.7, 36.1, 34.5, 30.4, 26.8, 26.4, 24.9, 16.8, 14.9, 11.5; HRMS: calculated for C_35_H_51_ClN_4_O_6_ ([M + H]^+^): 671.3586, found 671.3570.

**6z**: white powder; yield: 50.3%; mp: 120.1–121.5 °C; ^1^H NMR (400 MHz, CDCl_3_): δ (ppm) 9.02 (s, 1H), 7.49 (d, *J* = 8.4 Hz, 2H), 7.26 (d, *J* = 8.4 Hz, 2H), 6.48 (dd, *J* = 17.2, 11.2 Hz, 1H), 5.77 (d, *J* = 8.4 Hz, 1H), 5.31 (dd, *J* = 11.2, 1.2 Hz, 1H), 5.18 (dd, *J* = 17.2, 1.2 Hz, 1H), 4.79 (t, *J* = 5.6 Hz, 1H), 4.35 (d, *J* = 5.6 Hz, 2H), 3.78 − 3.74 (m, 4H), 3.41 (t, *J* = 5.0 Hz, 4H), 3.34 (dd, *J* = 9.6, 6.8 Hz, 1H), 3.21 − 3.02 (m, 4H), 2.63 − 2.58 (m, 4H), 2.58 − 2.45 (m, 4H), 2.36 − 2.01 (m, 6H), 1.89 (s, 1H), 1.76 (dd, *J* = 14.2, 2.4 Hz, 1H), 1.69 − 1.51 (m, 5H), 1.49 − 1.43 (m, 1H), 1.38 − 1.31 (m, 1H), 1.15 (s, 3H), 1.12 − 1.06 (m, 1H), 0.86 (d, *J* = 6.8 Hz, 3H), 0.70 (d, *J* = 6.8 Hz, 3H); ^13 ^C NMR (100 MHz, CDCl_3_): δ (ppm) 217.1, 168.9, 167.9, 157.4, 139.1, 136.6, 135.5, 128.5, 119.8, 117.3, 74.6, 68.5, 67.0, 59.8, 58.2, 53.8, 52.6, 45.5, 45.0, 44.5, 44.0, 43.7, 41.8, 36.7, 36.1, 34.5, 30.4, 29.7, 26.8, 26.5, 24.9, 16.7, 14.9, 11.5; HRMS: calculated for C_40_H_59_N_5_O_7_ ([M + H]^+^): 722.4487, found 722.4497.

### Biological assay

#### Bacterial solution preparation

Upon overall recovery of *S. aureus* ATCC 25923, MRSA ATCC 33591, MRSA ATCC 43300 and *E.coli* ATCC 25922, the single selected colony was inoculated in sterile Luria-Bertani (LB) broth and incubated at 37 °C for 1 8 ∼ 20 h. The bacterial solution was adjusted to 0.5 McFarland standard with saline and subsequently diluted with LB broth to the approximate concentration of 10^6^∼10^7^ CFU·mL^−1^.

#### MIC And MBC assays

MIC values were established *in vitro* using the agar dilution method^34^^,^^35^. Moreover, tiamulin fumarate was selected as the reference drug. All compounds were dissolved in a small amount of ethanol and diluted in distilled water to a concentration of 1280 µg·mL^−1^. Next, two-fold serially diluted pleuromutilin compound solutions containing distilled water were produced until the final concentration reached 0.625 µg·mL^−1^. After ten-fold dilution with LB broth, the two-fold serially diluted solutions were successively added to plate wells containing 10 ml of the above four bacterial solutions; then, solutions were incubated at 37 °C for 1 6 ∼ 18 h and data was recorded. Each procedure was repeated 3 times. MBC was considered when the compounds killed over 99% of the tested bacterial culture. MIC-corresponding wells and three previous wells were homogenised, serially diluted, and plated on Mueller-Hinton (MH) agar to determine MBC. Data was recorded after plates underwent incubation at 37 °C for 18–24 h. Each procedure was repeated 3 times.

#### Time-kill kinetics assay

Compounds **6p** and tiamulin fumarate were dissolved in MH broth to generate different solutions of 1 × MIC and 6 × MIC, followed by inoculation with the above bacteria suspension at 37 °C; After specified time intervals (0, 1, 2, 4, 6, 12 and 24 h), 10 µL of the above mixture was serially diluted 10-fold with saline and incubated at 37 °C for 24 h on MH agar plates. Following this, viable colonies were counted and expressed as log 10 CFU·mL^−1^. Each procedure was done a total of 3 times.

### CCK-8 assay

All cell lines were cultured in Dulbecco’s modified Eagle’s medium (DMEM), with 10% foetal bovine serum (FBS) and supplemented with 1% penicillin-streptomycin (P/S). The cell counting kit-8 (CCK*-*8) was purchased from Dojindo Laboratories (Kumamoto, Japan).

Briefly, a 100 μL suspension of 10000 LO2 cells and 20000 HEK293T cells were seeded in 96-well plates, respectively. They were treated with six different concentrations of **6p** (0.125 μg/mL, 0.25 μg/mL, 0.5 μg/mL, 1 μg/mL, 2 μg/mL and 4 μg/mL) and incubated at 37 °C in a CO_2_ incubator for 48 h. Afterwards, the CCK-8 solution (10 μL) was added to each well, and plates were incubated for an additional 1 h at 37 °C. The absorbance at 450 nm was then recorded using a SpectraMax-190 microplate reader (Molecular Devices, USA). Each procedure was done a total of 3 times.

### Molecular docking

The binding of synthesised compound **6p** was examined using the PTC ribosome complex and Tiamulin (PDB ID: 1XBP). In particular, the PTC ribosome model was constructed using residues within 30 Å from the binding site after extraction of the original ligand Tiamulin. In addition, both **6p** and the above ribosome model were prepared via AutoDockTools by removing water molecules, adding polar hydrogens, and assigning Gasteiger charges. Finally, the box (52.791, 122.679, 114.289) was established using original ligand as the grid centre with box size (36.758, 44.448, 36.761). Docking was performed by DOCK 6.9, and the 2 D interaction diagrams was generated via LigPlot^+^ v1.4^36^.

## Conclusions

In summary, a series of pleuromutilin derivatives possessing the piperazinyl urea linkage were designed and synthesised, and their antibacterial activities against Gram-positive and Gram-negative strains were evaluated. Nearly all synthesised compounds exhibited broad-spectrum antibacterial activities, and particularly, more potent activities against MRSA strains. Activities of **6p** were most effective with a MIC concentration of 0.1 2 5 ∼ 0.25 μg/mL, which was 8 ∼ 16 times that of Tiamulin. Time-kill kinetics indicated that at 1 × MIC, compound **6p** and Tiamulin had similar bactericidal effects against MRSA ATCC 33591; however compound **6p** was more effective than Tiamulin against *E.coli* ATCC 25922, since it eliminated total bacteria colony in 24 h while Tiamulin could not. Molecular docking demonstrated that aside from hydrophobic interactions, benzyl urea linkage and terminal nitro groups from the side chain could produce several hydrogen bonds and π-π stacking with surrounding residues.
